# pH-responsive antibodies for therapeutic applications

**DOI:** 10.1186/s12929-021-00709-7

**Published:** 2021-01-22

**Authors:** Tomasz Klaus, Sameer Deshmukh

**Affiliations:** Research and Development Department, Pure Biologics, Inc., Dunska 11, 54427 Wrocław, Poland

**Keywords:** Antibody generation, pH-responsive antibodies, Recycling antibodies, Sweeping antibodies, Tumor microenvironment, CAR-T cells

## Abstract

Therapeutic antibodies are instrumental in improving the treatment outcome for certain disease conditions. However, to enhance their efficacy and specificity, many efforts are continuously made. One of the approaches that are increasingly explored in this field are pH-responsive antibodies capable of binding target antigens in a pH-dependent manner. We reviewed suitability and examples of these antibodies that are functionally modulated by the tumor microenvironment. Provided in this review is an update about antigens targeted by pH-responsive, sweeping, and recycling antibodies. Applicability of the pH-responsive antibodies in the engineering of chimeric antigen receptor T-cells (CAR-T) and in improving drug delivery to the brain by the enhanced crossing of the blood–brain barrier is also discussed. The pH-responsive antibodies possess strong treatment potential. They emerge as next-generation programmable engineered biologic drugs that are active only within the targeted biological space. Thus, they are valuable in targeting acidified tumor microenvironment because of improved spatial persistence and reduced on-target off-tumor toxicities. We predict that the programmable pH-dependent antibodies become powerful tools in therapies of cancer.

## Background

From Pasteur [1] and Ehrlich [2] until today, an extraordinary scientific work has been put forth, enabling the clinical translation of monoclonal antibodies and antibody-based therapeutics for patients with unresolved clinical needs. Antibodies are a soluble form of B cell receptors (BCRs) and are essential molecules of the humoral immunity. Antibodies interact with the whole immune system through antibody-dependent cell-mediated cytotoxicity (ADCC), complement-dependent cytotoxicity (CDC), and antibody-dependent phagocytosis (ADCP) [3, 4].

Application of monoclonal antibodies for therapeutic purposes dates to 30 years. The unprecedented story of anti-CD3 Muromonab OKT3 clone [5–8] for immune modulation upon transplant and its approval by the Food and Drug Administration (FDA) has paved the way to success for several antibody-based therapeutics. To that extent, antibodies and antibody-based therapeutics have become one of the fastest-growing treatment modalities within the modern drug arsenal, with a projected revenue of 300B USD by 2025 [9]. As of March 2020, the FDA has approved over 90 antibodies, and several of them (> 10) are under review for a variety of disease indications. Many of them are focused on the treatment of various cancers [10].

Biological systems are complex and involve many protein–protein interactions and cellular metabolic processes. Cellular microenvironment manifests itself as an interplay of cellular energetics and protein–protein interactions. Thus, it provides a vast engineering landscape that can be exploited to alter either the protein function or a cellular metabolite and, eventually, cellular fate. Advances in antibody engineering through recombinant DNA technology have opened avenues that enable the control of biological processes in a space- and time-resolved manner. Antibodies can be endowed with properties to respond to a broad palette of environmental and physiological stimuli in cellular vicinities, such as pH. The ability to tap into the pH dependency of antibodies enables modulation of the cellular activity in a conditional and disease dependent manner. This aspect is vital to enhance the therapeutic potential conferred by traditional antibodies. Thus, on the cusp of the antibody engineering landscape, altering the activity of antibodies by generating pH-responsive antibodies is gaining momentum. Several groups have reported pH-responsive antibodies determining/altering the target protein function and hence the disease biology. The valid range of pH values in which such antibodies possess the potential to act as therapeutics spans the pH of acidified tumor microenvironment (TME, pH 5.9) and neutral human plasma (pH 7.4) [11].

**Fig. 1:**
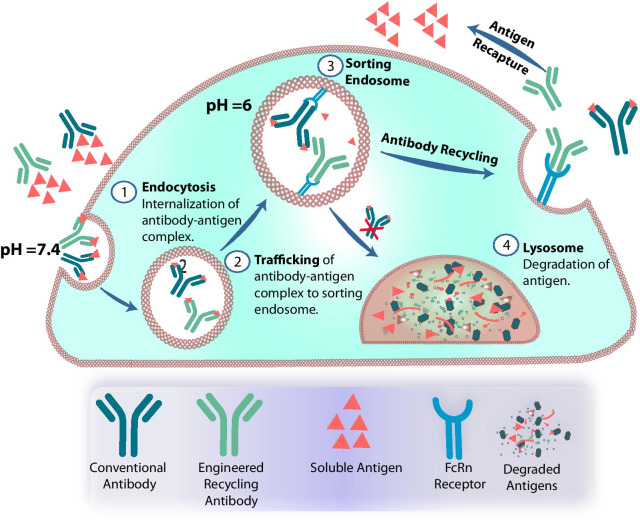
Schematic representation of trafficking mechanism for a conventional and recycling antibody. **a** Antibody–antigen complexes taken up by cells through non-specific pinocytosis or endocytosis are shuttled to sorting endosomes. In sorting endosome, the complex binds FcRn at acidic pH 6.0. A conventional antibody bound to an antigen at pH 6.0, is preferentially directed toward recycling pathways mediated by FcRn rather than transitioning from sorting endosome to the lysosome for lysosomal degradation. For an engineered recycling/sweeping antibody, reduced antigen affinity at pH 6.0 leads to dissociation of the antigen from the antibody–antigen complex. The dissociated antigen is trafficked towards the lysosome for degradation. Sweeping antibodies having a higher affinity to FcRn at pH 7.4 undergo FcRn mediated endocytosis. This higher affinity to FcRn leads to higher persistence of the antibody, and reduced availability of antigen

Antibodies are also internalized by cells and they reach early endosomes, where pH is about 6.5 [12]. In acidified endosomes, antibodies are captured by neonatal Fc receptor (FcRn) and recycled into extracellular space (Fig. 1). This phenomenon protects them from lysosomal degradation. Thus, therapeutic antibodies after administration into the human body encounter different microenvironments in which the concentration of H+ ions can differ by more than 30-fold. Several researchers have demonstrated that this difference is enough to create an antibody binding a cognate antigen exclusively at acidic or neutral pH [13, 14]. The range of pH values in the human body provides a window for engineering and designing of pH-responsive antibodies.

Here we review the recent advances made in pH-dependent antibodies focusing on recycling and sweeping antibodies. An update is provided on antigens targeted and the suitability of pH-responsive antibodies modulated by the TME. Applicability of pH-responsive antibodies in the engineering of chimeric antigen receptor T-cells (CAR-T) and in drug delivery to the brain is reviewed. Aspects of generation of such pH-responsive antibodies are also covered.

## Functionalities of antibodies targeting soluble or membrane-bound antigens

Based on the targeted antigen, therapeutic antibodies can be divided into three groups: (i) antibodies against soluble circulating targets, (ii) antibodies against membrane-bound targets with shedding, and (iii) antibodies against membrane-bound targets without shedding [15].

Shedding is a process mediated by membrane-anchored metalloproteases in which the extracellular domain of an antigen is released from the cell membrane [16]. Upon analysis of reported examples of pH-responsive antibodies, a clear correlation between the designed pH-dependent mode of action and the targeted antigen is observed. Antibodies against soluble antigens and shed membrane antigens are usually designed to bind their targets at neutral pH and release them at acidic pH. This approach allows the efficient elimination of these antigens from bodily fluids. In contrast, membrane-bound antigens associated with solid tumors are targeted mainly by antibodies engineered to recognize the antigen only at acidic pH. Acidic pH-selectivity allows for better spatial specificity and provides resistance to drug inactivation at low pH. Each of the mentioned categories of antibodies is considered in its class and discussed in the following sections.

## Targeting Soluble Antigens with Antibodies Releasing Antigen at Acidic pH

Immune complexes of polyclonal antibodies and a soluble antigen, which are formed during a typical immune response, are usually cleared by phagocytic cells. Depending on the antigen to antibody ratio, the complexes can be like soluble lattice or like large insoluble particles, which are deposited in filtrating tissues, e.g. kidneys. Immune complexes are also formed by therapeutic monoclonal antibodies and their targets. A typical monoclonal antibody can bind two antigen molecules at once. A lattice-like complex can be formed by a monoclonal antibody only if the antigen comprises at least two epitopes recognized by the antibody. The targets described in this section are monomeric soluble proteins; thus, their complexes with therapeutic antibodies are small and soluble.

In vivo, the administration of antibodies at times results in antibody-mediated antigen accumulation or, in other words, antibody buffering [17, 18]. Although this phenomenon is real for several clinically relevant antigens, it is limited in its context [19]. The concentration of an antigen in an extracellular fluid depends on the equilibrium between antigen production and its removal via endocytosis and lysosomal degradation. Administration of a specific antibody can profoundly increase the half-life of an antigen by trapping it in an antigen–antibody complex that is recycled by FcRn in endosomes. To address this issue, Igawa et al. developed antibodies that are pH-responsive and release bound antigen in acidified endosomes (Fig. 1) [20].

A conventional antibody with (sub)nanomolar affinity usually binds an antigen and remains in complex with the antigen for a long time. This effect results from a sought after mechanism for low dissociation rate during the development of biotherapeutics. In contrast to conventional antibodies, the molecules developed by Igawa et al. can release antigen upon internalization into endosomes. The antibodies work in cycles of antigen binding—endocytosis—antigen releasing—recycling into the extracellular fluid—binding the antigen again, and are called recycling antibodies [14] (Fig. 1).

Recycling antibodies can be further improved by increasing their internalization rate by enhancing their affinities towards a cell-membrane protein, e.g., FcRn [21] or FcγR2b [22]. Recycling antibodies with increased internalization rates are called sweeping antibodies [14]. Besides the pH-dependent variable region, well-characterized examples of sweeping antibodies comprised modified Fc-region that allow binding to the cell membrane [21, 22]. We expect also that the sweeping activity and increased internalization of an antibody can be achieved by the construction of a multispecific molecule that is equipped with a variable region recognizing one of the receptors recycled between plasma membrane and endosomes, e.g., insulin receptor, asialoglycoprotein receptor, high-mannose receptor, low-density lipoprotein-receptor or transferrin receptor.

The safety and efficacy of the sweeping antibodies was demonstrated in clinical trials with antibodies targeting complement component 5 (C5) [23–25]. C5 is cleaved by C5-convertase during complement activation cascade into two proteins: C5a—a chemoattractant for leukocytes and C5b that is involved in the formation of membrane attacking complex. The complex invades a cell membrane leading to its disruption and cell lysis. In several rare diseases, e.g., paroxysmal nocturnal hemoglobinuria or atypical hemolytic uremic syndrome, C5 is activated in an uncontrolled manner. Eculizumab—a monoclonal anti-C5 antibody that became a standard of care in the rare complement-dependent diseases, blocks the C5 cleavage. C5 concentration in human serum can reach more than 100 ug/mL [26]. Thus, to target C5 efficiently, very high doses of eculizumab must be administered. According to the prescribing information, the recommended dose of eculizumab for adult patients exceeds 1000 mg every two weeks. For comparison, a dose of adalimumab, an anti-tumor necrosis factor antibody used, e.g., in rheumatoid arthritis treatment, is about 40 mg per week. High and frequent intravenous administration of eculizumab increases the costs of therapy and reduces patient comfort.

Engineering the eculizumab antibody with a sweeping activity resolved the above-discussed challenges associated with this therapy. The introduction of histidine substitutions into its CDRs allowed pH-responsive antigen-binding leading to enhanced C5 clearance and prolonged half-life of the antibody. Moreover, additional mutations engineered in Fc-region enhanced the binding of the antibody to FcRn [27]. The generated antibody ALXN1210 (ravulizumab) was evaluated in clinical trials and was approved by FDA [28].

Another anti-C5 sweeping antibody SKY59, a humanized and engineered version for pH-responsiveness of a rabbit monoclonal antibody [29] was analyzed in preclinical animal models [30] and in phase I/II clinical trial [24, 31]. SKY59 showed long-lasting neutralization of C5, and it could inhibit C5 variant Arg885His that is not recognized by eculizumab [30].

A recent study showed the application of recycling and sweeping antibodies for the removal of toxins. The activity of conventional and pH-responsive variants of an antibody against Staphylococcal enterotoxin B (SEB) superantigen in a mouse model were compared [32]. Although all analyzed antibodies neutralized the toxin and reduced cytokine production, the pH-responsive molecules eliminated the toxin significantly faster than the conventional molecules. Recycling and sweeping antibodies theoretically can remove antigens out of circulation even if they do not neutralize them in in-vitro assays. Thus, they can be used as efficient antitoxins when a neutralizing antibody is not available.

Recycling and sweeping antibodies can target also extensively shed antigens. Bogen et al. recently developed a pH-responsive bispecific antibody targeting two crucial tumor markers CEACAM-5 and CEACAM-6 [33–35]. This unique molecule binds CEACAM-5 in a pH-responsive way and CEACAM-6 pH-independently. The presence of shed CEACAM-5 in the bloodstream hampers the efficacy of anti-CEACAM-5 antibody therapy. Administration of pH-dependent anti-CEACAM-5 antibody reduces the concentration of the antigen in serum and, consequently, allows for a better targeting of CEACAM-5 positive tumors. The bispecific antibody developed by Bogen et al. has not been tested in the animal models yet.

Sweeping antibodies allow better antigen clearance, or at least they suppress antigen accumulation, as demonstrated by targeting the soluble C5 antigen and the first bispecific antibody with putative recycling modality, which is still under development. The utility of recycling or sweeping antibodies might be limited in the case of targeting of the tumor microenvironment (TME). Low pH within TME prevents the binding of an antigen to the variable regions engineered to release it in acidified endosomes. Thus, in the next section, other strategies to target antigens within TME are discussed further.

## Targeting antigens within tumor microenvironment with acidic-pH-selective antibodies

### Importance of pH in tumor microenvironment

Hanahan and Weinberg [36, 37], in their seminal work, proposed that cancer is crafted by genetic alterations and disruption of cellular homeostasis. These alterations together lead to an extracellular milieu termed as tumor microenvironment (TME) and have several implications on tumor development and metastasis. A body of work has confirmed heterogeneity of TME, which is exacerbated by the somatic evolution of the malignancy [38–40]. Tumor acidosis resulting from alterations in the metabolism of tumor cells is a symbol of aberrant cell–cell interactions and the disruption of homeostasis [41, 42]. In this state, cells preferentially utilize glycolysis over oxidative phosphorylation as a primary means of energy liberation, an effect termed as anaerobic glycolysis [43]. Such a phenotype displays up to tenfold higher lactic acid load on the extracellular environment compared to intracellular compartment, leading to diffusive transport of H+ ions into interstitial space [44, 45].

pH is a globally pervasive parameter in TME. The success of therapies targeting the pH of the tumor microenvironment depends in part on the precise measurement of the tumor pH. Among recent advances made in the measurement of pH of TME there are techniques comprising PET radiotracers, MR spectroscopy, MRI, and optical imaging. Details about these techniques are covered in the reviews by Zhang et al. and Chen et al. [46, 47]. Changes of pH have an impact on the components of TME, such as stromal cells, extracellular matrix, and immune cells, contributing to immunosuppression, inflammation, immune escape, and disease progression. In acidic pH, effector immune cells (T & NK) undergo a state of reversible anergy followed by apoptosis, while suppressor myeloid lineage cells sustain tumor growth reducing drug response. These cells with differential functions often serve as a brake-on immune activity. They may also impede immunotherapy of the so-called cold-tumors i.e. tumors characterized by lack of infiltrating T cells as well as by lack of proinflammatory cytokines [48–50]. The extracellular acidity could also profoundly impact the bioavailability of therapeutic antibodies. On the other hand, acidic environment can be exploited as a necessary condition for activation of a therapeutic antibody. Precise spatiotemporal action of a therapeutic antibody is highly desired because it reduces drug toxicity. Examples of antibodies that are active only within acidic pH of TME are reviewed in the following section.

### Targeting TME by acidic pH-selective antibodies

Sulea et al. developed pH-dependent anti-human epidermal growth factor receptor 2 (HER2) antibodies that bind the antigen in acidic pH stronger than in a neutral environment. Kd value of the most pH-responsive antibody was about 290 nM at pH 7.3 and 6.6 nM at pH 5.6 [51]. The activity of the antibody was demonstrated in the tumor spheroid model. The antibody inhibited spheroid growth at pH 6.4, but the effect was not observed at pH 7.4. Trastuzumab, a control molecule in the experiment, inhibited spheroid growth at acidic and neutral pH. Trastuzumab is widely used in the treatment of HER2-positive breast cancer and HER2-positive metastatic gastric cancer. However, the clinical application of trastuzumab is associated with the risk of cardiotoxicity [52] because HER2 is expressed on adult cardiomyocytes. The development of acidic pH-selective antibodies might alleviate adverse effects by improving the targeting of TME and increasing the spatial specificity of the drugs.

Antibodies targeting tumors affect malignant cells via different Fc-dependent effector functions. Besides the direct effect on intracellular signaling upon antigen binding, an anti-tumor antibody usually activates cytotoxic cells expressing FcRs. Antibodies bound to a tumor cell activate also complement component C1q and, as a result, trigger complement cascade. It was shown that the binding of antibodies to C1q and FcRs depends on pH [53, 54]. Likely, the Fc region can also be modified to bind the specific receptors in a pH-dependent manner. This approach was suggested for the improvement of interaction between Fc fragment of IgG1 and FcγR3a expressed on natural killer cells [55].

Response to immunotherapy can be improved by raising pH in TME through bicarbonate taken orally [56]. This observation indicates that the action of immune checkpoint inhibitors is compromised by acidic TME, and the activity of common anti-CTLA-4 and anti-PD1 antibodies at low pH can be further improved. Moreover, the V-domain immunoglobulin suppressor of T cell activation (VISTA) was identified as a novel pH-dependent immune checkpoint [13]. VISTA was the first example of immune checkpoint activated exclusively in acidic TME. The extracellular domain of VISTA has an unusually extended loop comprising several histidine residues. At acidic pH, the loop binds a patch of sulfated tyrosines in P-selectin glycoprotein ligand-1 (PSGL-1). Acidic TME implies an active state of VISTA by keeping the histidine-rich loop in a positively charged state [13].

VISTA is highly expressed in myeloid cells, and it promotes the inhibitory function of myeloid-derived suppressor cells (MDSCs) in tumors [57]. Concurrently, PSGL-1 is expressed on T-cell, and it mediates extravasation of the cells into inflamed tissues. Tumor-infiltrating T-cells migrate into a more acidic environment where they encounter myeloid cells exposing activated VISTA. PSGL-1 on the infiltrating T-cells forms complex with VISTA on the myeloid cells. Therefore, the T cell immune response is inhibited [13].

VISTA-mediated immunosuppression was reversed by antibodies blocking the interaction between VISTA and PSGL-1 in vivo. A comparison of the efficacy of acidic-pH-selective and conventional VISTA-blocking antibodies revealed superior pharmacokinetics of the pH-responsive molecule. VISTA is expressed on circulating and organ-resident myeloid cells. Thus, the conventional anti-VISTA antibody accumulated in leukocyte-rich organs. In contrast, the acidic pH-selective antibody localized primarily within the tumor and exhibited prolonged blood mean residence time in animal models [13].

The presented examples demonstrate that pH-responsive antibodies targeting tumors need to be screened for activity at the low pH typical for TME. The engineering of antibodies should not be limited to searching for molecules with activity within a broad range of pH values. Acidic-pH selectivity might substantially improve spatial specificity of biotherapeutics.

### Targeting TME with acidic-pH-selective CAR-T cells

**Fig. 2:**
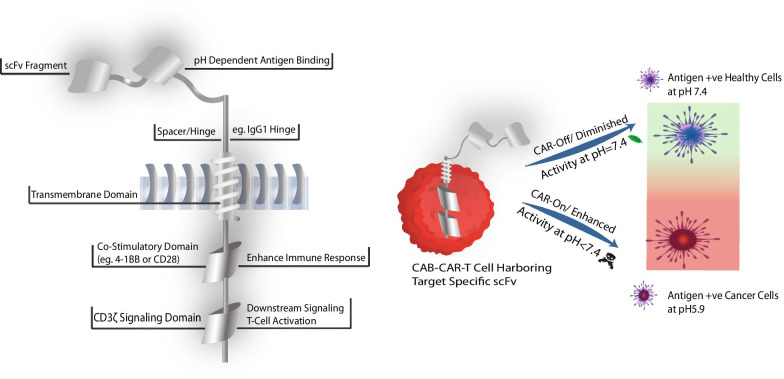
Schematics of a recombinant CAR-T cell. **a** Construct comprises a downstream signaling domain for T-cell activation; a co-stimulatory domain that enhances cytokine production; transmembrane domain traversing the cellular membrane for anchoring the CAR; spacer region affecting the flexibility and functionality; scFv: a targeting domain recognizes the tumor-associated antigen. **b** Schematics for a conditionally active biologics instilling “AND” logic gate characteristics to the CAR-T cell are shown. A pH-dependent scFv is engineered for binding to its cognate receptor on tumor cells with high affinity only within TME at pH < 7.4, i.e. “CAR-On” mode. In the context of antigens expressed on healthy cells (at pH 7.4) the affinity to cognate antigens is severely diminished i.e. “CAR-Off” mode thus sparing the normal/healthy cells

Variable fragments of pH-responsive antibodies can be used as targeting domains in chimeric antigen receptors (CARs, Fig. 2). T cells engineered to express CARs (CAR-T cells) are one of the most attractive fields for cancer therapeutics. CARs are analogous in action to T cell receptors [58, 59]. CARs are composed of single chain variable fragment (scFv) of an antibody for recognition of the malignant cells, spacers, transmembrane domain, and intracellular domains for enhanced immune response and T-cell downstream activation. Currently, this treatment modality has demonstrated unprecedented response rates of 70–90% in B-cell malignancies with two FDA approvals [60–62]. However, this treatment is not a panacea. CAR-T cells can elicit a robust immune response, which can lead to potentially fatal inflammatory reactions like cytokine release syndrome [63]. Cytokines released at an acceptable limit suggests the efficacy of the treatment, while severe cases lead to fatalities, as demonstrated in several clinical trials [64], e.g., affinity-enhanced TCR against the melanoma-associated antigen 3 (MAGE-A3) trial [65]. Unfortunately, the success of CAR-T cells is obscured in treating solid tumors [66, 67]. It is attributed to lack of targetable antigens expressed exclusively on tumor cells. This leads to on-target off-tumor cross-reactive toxicities and illustrates that for the CAR-T therapy, precision in detecting cancer could be improved [67–72].

Molecular recognition and precision of therapy have been enhanced by providing additional functionality to CAR-T cells, thereby orthogonally modulating their activation as per their environment. Some novel ways comprise using inhibitory receptors, among them novel synthetic variants of Notch receptors, logical CARs, which are equipped with conditional activation modules [73–75]. There are several excellent reviews on CAR-T cells and their applications in therapy [76–79]. Hence additional details are not included here. Instead, the emphasis is given on spatial activation and localization of CAR-T-cells within TME. Moreover, examples of engineered CAR-T cells modulated by the difference in pH within TME [80, 81] are also discussed.

In one of the critical studies, it was confirmed that the modulation of T cell metabolism could alter the cell function [82]. Glycolytic metabolite phosphoenolpyruvate (PEP) maintains signaling and effector function of the nuclear factor of activated T cells (NFAT). Insufficient PEP levels can cause anti-tumor T cell responses to diminish. In the same study, it was demonstrated that overexpression of PEP carboxykinase 1 catalyzes the production of PEP in T cells, which leads to enhanced effector function [82]. Another example from a patent shows pH alteration in TME of the CHO xenograft tumor model and a decrease in cathepsin activity upon administration of sodium bicarbonate [83]. The relative protease activity of cathepsin is higher at lower pH and lower at higher pH and serves as a indirect measure of the pH of the tumor microenvironment. Administration of sodium bicarbonate was further used to verify and modulate the activity of CAR-T-cells *in-vivo* by shifting the pH within the TME. Given nutrients deficits in solid tumors, modulation of CAR-T cell’s metabolic characteristics is critical for effective therapy.

F1 Oncology Inc./BioAtla, in their patent application [84], extended their proprietary conditionally active biologics (CAB) platform to develop a novel approach for CAR-T therapy. CAB platform is utilized to discover antibodies that are activated or inactivated under specified physiological conditions depending on the cellular microenvironment. A schematic is represented in Fig. 2.

AXL receptor tyrosine kinase (AXL) and receptor tyrosine kinase like orphan receptor 2 (ROR2) are cancer-associated antigens. Elevated expression levels of these receptors are observed in various cancers of high unmet clinical needs [85, 86]. CARs harboring anti-AXL and anti-ROR2 scFv were engineered to provide pH-dependent binding functionality, resulting in CAB-CARs. It was demonstrated that in TME under acidic conditions (pH ~ 6.7), the affinity of the CAB-CAR scFv domains against their cognate antigen was higher as compared to the pH at 7.4. Therefore, tumor target recognition via these scFvs and eventually T cells transfected with such CARs become activated only within TME in a pH-dependent manner. At the same time, on-target off-tumor toxicity was reduced. These CAB-CAR-T cells displayed reversible “AND” logic gate properties, requiring both antigen presence and TME conditions for activity.

In the same patent application, multispecific pH-dependent CAB-CAR T cells were reported. One of the antigen-binding domains is specific to either ROR2 or AXL. The second antigen-binding domain binds another target, e.g., other antigen associated with cancer (CD19, CD38, HER2, EGFR, CEA, or IL-13R-a2), or a cancer-related ligand (IL-13, heregulin, VEGF).

Furthermore, it was shown that T cells transduced with the anti-AXL CAB-CAR or the anti-ROR2 CAB-CAR constructs elicited conditional cytokine secretion and activation of these T cells upon exposure to their cognate tumor antigens, in a pH-dependent manner. The readout was measured as higher levels of IL-2, IFN-γ, early activation marker CD69, and the degranulation marker CD107a at pH 6.7 but not at pH of 7.4. Additionally, these CAB-CARs were shown to be efficacious in a preclinical mouse model demonstrating the utility of the approach in accessing solid tumors and driving cytolysis of target cells.

## pH-responsive antibodies with improved pharmacokinetics or pharmacodynamics

Besides removing soluble proteins from circulation and targeting TME, pH-responsive modality might improve the pharmacokinetics of antibodies that exhibit target mediated clearance. Well-known examples of this phenomenon are antibodies binding proprotein convertase subtilisin/Kexin type 9 (PCSK9) that are quickly cleared from the circulation [87, 88]. Substantial improvement in the pharmacokinetics of anti-PCSK9 antibodies was achieved with their recycling variants, i.e., antibodies binding the antigen at neutral pH and releasing it in acidified endosomes. The recycling variants may enable less frequent or lower dosing schemes of the anti-PCSK9 antibodies [87].

A short half-life of molecules can be prolonged by fusing them with a protein preventing their clearance by FcRn-dependent mechanism, e.g., albumin [89]. Qiu et al. developed two acidic pH-selective anti-FcRn scFvs that might be used as moieties extending half-life of, e.g., therapeutic peptides [90]. The scFvs bind FcRn at acidic pH only. Thus, they mimic albumin behavior and enable the recycling of a molecule that was fused with them.

Application of recycling antibodies in tumor targeting seems to be counterintuitive since recycling antibodies do not bind their cognate antigens in acidic pH, which is the hallmark of TME. However, a recycling variant of therapeutic anti-HER2 antibody conjugated with a cytotoxic drug showed better cytotoxicity toward HER2-positive tumors than the non-recycling variant [91]. The recycling modality allowed better lysosomal delivery of the drug that was crucial for the efficacy of the antibody–drug conjugate.

Also, other pH-dependent antibodies target cytotoxic T-lymphocyte-associated antigen 4 (CTLA-4) on T-cells present in TME. CTLA-4 is an inhibitory receptor acting as a major negative regulator of T-cells. CTLA-4 shares B7-family ligands with stimulatory receptor CD28, but CTLA-4 binds the ligands considerably stronger. Ipilimumab, the first anti-CTLA-4 immune checkpoint inhibitor, was approved by the FDA for melanoma treatment in 2011 [92]. However, the antibody demonstrates severe immunotherapy related adverse effects. Advances in the understanding of CTLA-4 biology and intracellular trafficking lead to new ideas about how CTLA-4 can be safely exploited as a target for immunotherapy [93–95].

CTLA-4 is recycled between the plasma membrane and endosomes by binding to lipopolysaccharide-responsive and beige-like anchor protein (LRBA) [93, 95]. Antibodies that bind CTLA-4 disrupt the recycling process. Consequently, CTLA-4 is systemically directed to lysosomal degradation, and autoimmunity-related adverse effects are developed due to the unstoppable action of cytotoxic T cells. Conversely, pH-dependent anti-CTLA-4 antibodies, which dissociate from the target under acidic pH in endosomes, allow physiological CTLA-4 recycling, and reduce adverse effects. The mode of action of the pH-dependent anti-CTLA-4 antibodies is rather counterintuitive because CTLA-4 is a membrane antigen that needs to be inactivated on T-cells within acidic TME. However, the antibodies demonstrated superior efficacy in a humanized mouse model [93].

## pH-responsive antibodies in crossing the blood–brain barrier

Conventionally, central nervous system (CNS) is pursued as immune-privileged [96]. However, there are reports [97, 98] suggesting the immune system interfaces with the brain. The poor transport of active ingredients including monoclonal antibodies, antibody–drug conjugates (ADCs), and hydrophilic substances across the blood–brain barrier (BBB) impedes the development of new therapies to the clinic. Hence, the prognosis for patients with CNS diseases remains bleak. BBB is a dynamic and protective neurovascular unit, the functionality of which depends on a close interplay between various cells, receptors, enzymes, and transporters [99]. For the normal functioning of the brain, BBB allows the passage of specific molecules based on different mechanisms of transport [100]. Broadly, substances can undergo simple diffusion, facilitated diffusion, carrier-mediated transport, receptor-mediated transcytosis/endocytosis, absorptive-mediated transport, and carrier-mediated efflux to pass through the BBB. The transport mechanisms have been discussed in thorough detail elsewhere [100]. For large molecules like antibodies and ADCs, the primary pathway to cross BBB is receptor-mediated transport (RMT).

RMT is specific to a receptor expressed on the endothelial cells. Ligands targeting their cognate antigens like transferrin, insulin, insulin-like growth factor I and II, angiotensin II [101, 102] have been engineered to deliver drugs across the BBB in the form of protein-drug conjugates. These ligand-antigen interactions facilitate transcytosis, one of the mechanisms for pH-dependent recognition. A recent reviews of antibodies targeting the blood–brain barrier as well as CNS diseases, particularly glioma, was published [102–104]. Therefore, examples in this review are only incorporated to cater to pH-dependent properties of antibodies.

Transferrin receptor (TfR) has been studied as an internalizing receptor on endothelial cells in the BBB [105]. ADCs with corresponding anti-TfR antibody binding TfR on the apical side of the BBB have been demonstrated to deliver payloads into the brain [106–110]. A method termed “Trojan Horse” is utilized, wherein the antibody targets an epitope that is distinct from the ligand-binding site on the receptor. The ligand-receptor complex then undergoes internalization by endocytosis and leads to the formation of intracellular trafficking vesicles [111]. Inside the endosome, due to pH change from 7.4 to 6.5, the ligand is released from the receptor-ligand complex to exert effects in the brain [112].

Up to 90% of anti-TfR antibody clone OX26 and 8D3 with a high affinity to their cognate antigen were found in the brain capillaries upon intravenous administration at the timepoint of 24 h; in contrast, the low-affinity antibodies detected at the brain parenchyma were co-localized with a neuronal marker [113]. Due to prolonged residence time, the degradation of the high-affinity antibody-TfR complex occurred mainly in lysosomes in contrast to the low-affinity complex, which is congruent with the in vivo observation leading to low brain exposure. As an alternative improvement approach, engineering of the antibody for monovalency was reported [114].

Long residence time, affinity, and effect of an antibody can be optimized for appropriate brain exposure by engineering pH-responsive antigen-binding property to the antibody, thereby improving its transcytosis. Antibodies with reduced affinity to TfR at pH 5.5, as compared to affinity at pH 7.4, were shown to have greater transcytosis into the brain than antibodies that have similar binding affinity at both pH 5.5 and 7.4 [115].

A similar strategy was reported in the patent WO2012143379A1. A fusion polypeptide with a binding site to an internalizing receptor was disclosed. The anti-TfR antibody MEM-189 with reduced affinity at acidic pH could undergo transcytosis and recycling. In another patent, UW-Madison researchers have disclosed an anti-TfR scFv displaying higher dissociation at pH 5.5 than at a physiological pH of 7.4 [116]. A comparison of this antibody and its parental clone revealed differential trafficking and up to 2.6-times higher intracellular accumulation of the pH-responsive molecule. 

Although RMT has been studied extensively for delivery of drugs into the brain, forwarding antibodies exploiting RMT to the clinic has been a daunting task [112]. Low efficiency of the antibody delivery across BBB, degradation within endosomes, as well as antibody trapping in the endothelial cells contribute to the insufficient localization of the therapeutic molecule in the brain. The un-differentiated expression of TfR and similar receptors in various tissues could potentially elicit on-target off-tumor toxicity. The engineering of the pH-responsiveness to the antibodies may help with balancing affinity and desired releasing of biotherapeutics targeting the brain tissue.

## Generation of pH-responsive antibodies

### Generation of pH-responsive variable domains

Published examples of the generation of pH-responsive antibodies demonstrated different methodologies, but an overall strategy for many of the known pH-responsive antibodies was based on the engineering of a pre-existing specific binder. pH-responsive variants were engineered from parental molecules, including therapeutic antibodies [27, 51], binders selected using immune libraries of displayed antibodies [33], human antibodies derived from transgenic animals[13], or even rabbit monoclonal antibody [29].

Almost all known pH-responsive antibodies sense pH due to histidine residues within their variable regions. pKa value of the histidine side chain is about 6; thus, at pH below 6.0, the histidine side chain is mostly protonated, whereas, at physiologic pH 7.4, it is deprotonated. It was shown that an increased number of ionizable groups correlates with stronger pH-dependency [33, 117]. Since histidine is rare within germline and matured sequences of CDRs in antibodies, synthetic or semi-synthetic repertoires of histidine doped variants can be screened for pH-responsive binders [118]. There are also reports describing the generation of mouse pH-responsive antibodies by hybridoma technique, in which B lymphocytes isolated from immunized mice were immortalized by fusion with myeloma cells [119, 120]. However, screening of naïve repertoires, even if they are artificially doped with histidine, is usually laborious, and only a few percent of the identified clones are pH-dependent [119, 121]. We found only one report describing panning of a naïve phage-displayed repertoire, in which 50% of unique clones bound target selectively at acidic pH [90]. Protocols for the generation of pH-responsive binders from a naïve repertoire are available [118].

Also, pH value may influence the state of an antigen, and pH-selective antibodies can be raised against the state of the antigen. Epitopes that depend on pH were identified in C5 [30] and VISTA [13]. In these examples, the epitopes comprised three histidine residues. Antibodies binding these epitopes were generated and engineered to be pH selective. Histidine residues in the epitopes partially determined the pH-selectivity of the antibodies as demonstrated by solving crystal structures of antigen–antibody complexes and loss-of-function mutagenesis of the antigen [13, 30].

As we explained above, the most successful approach to generate a pH-responsive antibody is the engineering of a pre-existing specific binder. Researchers usually combine different methods of protein engineering, based on the rational design as well as on screening of large libraries of displayed variants.

Rational design based on sequence or structure analysis was applied for well-characterized antibodies, e.g., eculizumab [27], pertuzumab [91], and the equivalent of trastuzumab [51]. Rational design requires many input data; thus, it can be applied only to scrutinized molecules. At the beginning, histidine-scanning is usually employed to find first leading molecules with preferred binding characteristics. Histidine scanning is a variant of the well-known alanine scanning, where selected residues in a protein are mutated, and then functional analysis of the mutein is performed. In the case of eculizumab, a small library of 66 variants was created by replacing each position within CDRs by histidine [27]. In other examples, 20 variants of anti-SEB antibody 3E2 were designed as yeast displayed scFvs, but only nine of them were successfully expressed, suggesting that introduced histidine affected folding [32]. Some properties of mutants can be predicted with reasonable accuracy by computational methods. Sulea et al. performed in silico histidine-scanning of anti-HER2 antibody [51]. The analysis was based on the crystal structure of the antigen–antibody complex. Then, the authors selected variants that met applied criteria and expressed them to analyze their properties. This approach reduced the number of initial binders for experimental evaluation.

Display technologies allowed screening of vast repertoires of variants and were applied as an alternative or complementary way for identification of leading pH-responsive variants of an antibody. Different types of the displayed libraries were reported: simple phage-displayed scFv libraries of pertuzumab variants with NNB-randomized CDRs [91]; yeast-displayed libraries of separately synthesized genes encoding anti-VISTA antibody variants with charged residues introduced into CDRs [13]; yeast-displayed libraries of histidine-doped light chain paired with anti-CEACAM-5 VH-only binder [33]. All the libraries allowed to select pH-responsive variants of the parental molecules. The libraries were subjected to positive and negative steps by incubation with the antigen at predetermined pH, then desired pools of variants were retrieved, amplified, and introduced as input for subsequent selection round [13, 33, 91].

The rational design or selection of a displayed library was followed by screening. Typically, the binding of each variant to antigen was analyzed in a simple ELISA at two different pH, e.g., 5.8 and 7.4 [29, 91]. A comparison of the signal obtained in the two pH values facilitated initial selection of promising hits. More detailed analyzes based on surface plasmon resonance or biolayer interferometry were done in parallel with ELISA [27, 29]. Some authors also did cell-based assays at different pH at a very early stage of biotherapeutic development [13, 32]. Then, selected leading variants were further engineered. Identified mutations promoting pH-responsive binding were combined, and their impact was re-evaluated in biophysical assays. An additive effect of combined mutations was reported by at least three independent groups [13, 29, 33].

### pH-responsive Fc and its engineering

Fc region determines the effector functions of an antibody and its persistence in the blood. Thus, the desired modes of action of an antibody can be achieved by selecting appropriate Fc isotype. Most antibodies available in the clinic today belong to IgG1 or IgG4 subclasses. IgG1 has potent effector functions like activation of ADCC and complement cascade. Additionally, the pharmacokinetics of IgG1 is suitable for translation to the clinic. IgG2 and IgG4 are weak activators of the effector functions. Human IgG3 has not been used as a scaffold in any clinically approved biologics because of difficulties in its development [122]. However, IgG1/IgG3 heterodimeric variants are reported to have a higher cytotoxic potential as compared to wild-type counterparts [123]. The introduction of point mutations into Fc-region leads to precise control of the effector functions and half-life of an antibody. Comprehensive reviews of mutations within Fc and their impact on IgG1 characteristics were written by Kang and Jung [124], Ward, and Ober [125], Igawa et al. [14], and Bruhns and Jönsson [126]. These papers cover all the recent advances in Fc-engineering.

Interaction between Fc and FcRn was a prototype of pH-selective variable regions. FcRn binds exposed loops between CH2 and CH3 domains of heavy chain in IgG. Stoichiometry of FcRn and IgG interaction is in 2:1 [127]. The loops within the CH2/CH3 interface comprise histidine residues that change their protonation state depending on pH [128]. FcRn binds Fc only when the histidine residues within the CH_2_/CH_3_ interface are protonated. The slightly acidic environment within early endosomes allows IgG binding to FcRn, and consequently, IgG is salvaged from lysosomal degradation.

In contrast to conventional antibodies, sweeping antibodies were modified to bind FcRn at neutral pH [14]. FcRn is ubiquitously expressed on many cells; therefore, it provides extended adsorption surface for immune complexes. Binding to FcRn at neutral pH combined with the recycling activity of the variable region increased antigen clearance from circulation [21]. An alternative to FcRn-based sweeping was FcγR2b-based sweeping [22]. Mutations within the Fc region, allowing both types of sweeping, were reviewed previously [14]. Similar mutations in the FcRn binding interface on IgG were described by Vaccaro et al. in so-called AbDegs (antibodies that enhance IgG degradation) [129]. However, it has not been verified if they can be used for generation of sweeping antibodies.

Increasing Fc affinity to FcRn at neutral pH was frequently associated with reducing the persistence of a biotherapeutic in blood. This issue was solved by modulation of Fc affinity to FcRn to preserve sweeping activity and desired antibody half-life [119]. Another solution was the generation of FcγR2b-based sweeping [22]. The most recent approach based on multispecific molecules opens new ways for the generation of sweeping antibodies with long blood persistence [33]. Nevertheless, this approach must be verified in animal models to confirm its putative sweeping activity.

**Fig. 3:**
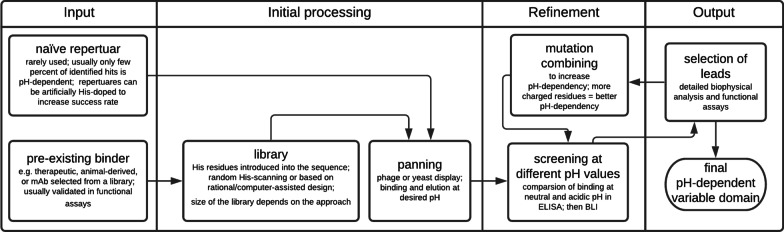
Generation of pH-dependent variable domain

The generation of pH-responsive antibody is usually a multi-step and laborious work (Fig. 3). The growing body of examples of pH-responsive molecules proved that it is achievable even by basic protein engineering laboratories equipped with instruments for affinity measurements. The pH-responsive mode of action opens new avenues to improve biotherapeutics. We expect that other methods of protein engineering enter this field, e.g., site-specific chemical modifications of proteins. The first pH on–off binding switch antibodies were generated by nitration of the tyrosine side chain in 1994 [130]. The report predicted that the most innovative solutions would be developed by combining recombinant and chemical technologies. We believe that this approach is ready to be verified in the development of new biotherapeutics.

## Conclusions

Molecular heterogeneity of the TME renders it amenable to novel therapeutic molecules. This heterogeneity manifests itself as an opportunity for the development of a highly specific, customizable, and efficacious therapeutic regime. Antibody-based therapies are an exciting and revolutionary treatment modality targeting previously undruggable diseases and antigens within TME. Nevertheless, side effects like off-target toxicities need addressing. Thus, high importance is currently given to engineering next generation of drugs for precise spatiotemporal control and inducible gain of function within the targeted biological space. For antibodies it may be achieved via engineering pH-sensitive motifs in their variable regions.

Soluble antigens can be efficiently removed out of the circulation using recycling and sweeping antibodies. A recycling antibody dissociates the antigens in sorting endosomes, where pH is acidic. The dissociated antibody is then recycled back to the plasma while the antigen is degraded in the lysosome. A sweeping antibody has also engineered Fc to enhance internalization rate of the antibody-antigen complex and eventually to increase antigen degradation in lysosomes.

The spatial persistence of an antibody confined to tumor vicinity is interesting to restrict its activity and localization, especially for highly differentiated TME. This strategy was demonstrated using monoclonal antibodies and CAR-T cells based on “AND” logic with variable domain engineered for higher target binding at acidic pH. The logic gate approach improves the localization of an antibody or CAR-T cells and it reduces the off-tumor toxicity. Platforms based on phage or yeast display combined with standard protein engineering techniques can be utilized for generating pH-responsive antibodies. The developability aspect of such antibodies, needs to be carefully considered at an early stage of binder identification. This consideration enables facile manufacturing strategy and a faster route to the clinic.

pH-sensitive antibodies possess strong therapeutic potential. These antibodies should be further explored either as a standalone treatment modality or as a part of next-generation biological therapies having multiplexed effector modules for a specific biological readout. Such programmable antibody-based therapies can be a powerful tool in the physician’s arsenal.

## Data Availability

Not applicable.

## Abbreviations

ADCC: Antibody-dependent cell-mediated cytotoxicity
ADCs: Antibody–drug conjugates
AXL: Tyrosine-protein kinase receptor UFO
BBB: Blood–brain barrier
C5: Complement component 5
CAB: Conditionally active biologics
CAR: Chimeric antigen receptor
CAR-T: Chimeric antigen receptor T cells
CD: Cluster of differentiation
CDRs: Complementarity-determining regions
CEACAM: Carcinoembryonic antigen-related cell adhesion molecule
CHO: Chinese hamster ovary cell
CNS: Central nervous system
CTLA-4: Cytotoxic T-lymphocyte-associated antigen 4
Fc: Fragment crystallizable region
FcRn: Neonatal Fc receptor
FcRs: Fc-receptors
FcγR2b: Fc gamma receptor 2b
FDA: Food and Drug Administration
HER2: Human epidermal growth factor receptor 2
NK: Natural killer
PCSK9: Proprotein convertase subtilisin/kexin type 9
scFv: Single chain variable fragment

## References

[1] Pillot J (1996). The year of Pasteur: from the concept of antibody and antigen by Bordet (1895) to the ELISA. What future for immunological diagnosis?. Clin Diagn Virol.

[2] Lindenmann J (1984). Senior overviews. Scand J Immunol.

[3] Griggs J, Zinkewich-Peotti K (2009). The state of the art: immune-mediated mechanisms of monoclonal antibodies in cancer therapy. Br J Cancer.

[4] Zahavi D, AlDeghaither D, O’Connell A, Weiner LM (2018). Enhancing antibody-dependent cell-mediated cytotoxicity: a strategy for improving antibody-based immunotherapy. Antibody Ther.

[5] Van Wauwe JP, De Mey JR, Goossens JG (1980). OKT3: a monoclonal anti-human T lymphocyte antibody with potent mitogenic properties. J Immunol.

[6] Kung P, Goldstein G, Reinherz EL, Schlossman SF (1979). Monoclonal antibodies defining distinctive human T cell surface antigens. Science.

[7] Kubo RT, Born W, Kappler JW, Marrack P, Pigeon M (1989). Characterization of a monoclonal antibody which detects all murine alpha beta T cell receptors. J Immunol.

[8] Chatenoud L (2003). CD3-specific antibody-induced active tolerance: from bench to bedside. Nat Rev Immunol.

[9] Lu R-M, Hwang Y-C, Liu I-J, Lee C-C, Tsai H-Z, Li H-J (2020). Development of therapeutic antibodies for the treatment of diseases. J Biomed Sci.

[10] Kaplon H, Muralidharan M, Schneider Z, Reichert JM (2020). Antibodies to watch in 2020. MAbs.

[11] Wike-Hooley JL, Haveman J, Reinhold HS (1984). The relevance of tumour pH to the treatment of malignant disease. Radiother Oncol.

[12] Hu Y-B, Dammer EB, Ren R-J, Wang G (2015). The endosomal-lysosomal system: from acidification and cargo sorting to neurodegeneration. Transl Neurodegen.

[13] Johnston RJ, Su LJ, Pinckney J, Critton D, Boyer E, Krishnakumar A (2019). VISTA is an acidic pH-selective ligand for PSGL-1. Nature.

[14] Igawa T, Haraya K, Hattori K (2016). Sweeping antibody as a novel therapeutic antibody modality capable of eliminating soluble antigens from circulation. Immunol Rev.

[15] Kuang B, King L, Wang HF (2010). Therapeutic monoclonal antibody concentration monitoring: free or total?. Bioanalysis.

[16] Mezyk R, Bzowska M, Bereta J (2003). Structure and functions of tumor necrosis factor-alpha converting enzyme. Acta Biochim Pol.

[17] Byrd JC, O’Brien S, Flinn IW, Kipps TJ, Weiss M, Rai K (2007). Phase 1 study of lumiliximab with detailed pharmacokinetic and pharmacodynamic measurements in patients with relapsed or refractory chronic lymphocytic leukemia. Clin Cancer Res.

[18] Haringman JJ, Gerlag DM, Smeets TJM, Baeten D, van den Bosch F, Bresnihan B (2006). A randomized controlled trial with an anti-CCL2 (anti–monocyte chemotactic protein 1) monoclonal antibody in patients with rheumatoid arthritis. Arthritis Rheum.

[19] Milgrom H, Fick RB, Su JQ, Reimann JD, Bush RK, Watrous ML (1999). Treatment of allergic asthma with monoclonal anti-IgE antibody. N Engl J Med.

[20] Igawa T, Ishii S, Tachibana T, Maeda A, Higuchi Y, Shimaoka S (2010). Antibody recycling by engineered pH-dependent antigen binding improves the duration of antigen neutralization. Nat Biotechnol.

[21] Igawa T, Maeda A, Haraya K, Tachibana T, Iwayanagi Y, Mimoto F (2013). Engineered monoclonal antibody with novel antigen-sweeping activity in vivo. PLoS ONE.

[22] Iwayanagi Y, Igawa T, Maeda A, Haraya K, Wada NA, Shibahara N (2015). Inhibitory FcγRIIb-mediated soluble antigen clearance from plasma by a pH-dependent antigen-binding antibody and its enhancement by Fc engineering. J Immunol.

[23] ALXN1210 (Ravulizumab) Versus Eculizumab in Complement Inhibitor Treatment-Naïve Adult Participants With Paroxysmal Nocturnal Hemoglobinuria (PNH) - Full Text View - ClinicalTrials.gov n.d. https://clinicaltrials.gov/ct2/show/NCT02946463 accessed March 27, 2020

[24] Dec 06,2018 | Chugai Presents Interim Analysis Data of Phase I/II Study of SKY59, anti-C5 antibody in PNH at ASH | News. CHUGAI PHARMACEUTICAL CO, LTD n.d. http://www.chugai-pharm.co.jp/english/news/detail/20181206170001_572.html accessed March 27, 2020

[25] A Phase 3, Open-Label Study of ALXN1210 in Children and Adolescents With Paroxysmal Nocturnal Hemoglobinuria (PNH) - Full Text View - ClinicalTrials.gov n.d. https://clinicaltrials.gov/ct2/show/NCT03406507 accessed March 27, 2020

[26] Corvillo F, García-Morato MB, Nozal P, Garrido S, Tortajada A, de Córdoba SR (2016). Serum properdin consumption as a biomarker of C5 convertase dysregulation in C3 glomerulopathy. Clin Exp Immunol.

[27] Sheridan D, Yu Z-X, Zhang Y, Patel R, Sun F, Lasaro MA (2018). Design and preclinical characterization of ALXN1210: A novel anti-C5 antibody with extended duration of action. PLoS ONE.

[28] FDA approves ravulizumab-cwvz for paroxysmal nocturnal hemoglobinuria. FDA 2019. https://www.fda.gov/drugs/resources-information-approved-drugs/fda-approves-ravulizumab-cwvz-paroxysmal-nocturnal-hemoglobinuria

[29] Sampei Z, Haraya K, Tachibana T, Fukuzawa T, Shida-Kawazoe M, Gan SW (2018). Antibody engineering to generate SKY59, a long-acting anti-C5 recycling antibody. PLoS ONE.

[30] Fukuzawa T, Sampei Z, Haraya K, Ruike Y, Shida-Kawazoe M, Shimizu Y (2017). Long lasting neutralization of C5 by SKY59, a novel recycling antibody, is a potential therapy for complement-mediated diseases. Sci Rep.

[31] Röth A, Egyed M, Ichikawa S, Kim JS, Nagy Z, Gaàl Weisinger J (2018). The SMART anti-hC5 antibody (SKY59/RO7112689) shows good safety and efficacy in patients with paroxysmal nocturnal hemoglobinuria (PNH). Blood.

[32] Kroetsch A, Qiao C, Heavey M, Guo L, Shah DK, Park S (2019). Engineered pH-dependent recycling antibodies enhance elimination of Staphylococcal enterotoxin B superantigen in mice. MAbs.

[33] Bogen JP, Hinz SC, Grzeschik J, Ebenig A, Krah S, Zielonka S (2019). Dual function pH responsive bispecific antibodies for tumor targeting and antigen depletion in plasma. Front Immunol.

[34] Blumenthal RD, Leon E, Hansen HJ, Goldenberg DM (2007). Expression patterns of CEACAM5 and CEACAM6 in primary and metastatic cancers. BMC Cancer.

[35] Lee JH, Lee S-W (2017). The roles of carcinoembryonic antigen in liver metastasis and therapeutic approaches. Gastroenterol Res Pract.

[36] Hanahan D, Weinberg RA (2000). The Hallmarks of cancer. Cell.

[37] Hanahan D, Weinberg RA (2011). Hallmarks of cancer: the next generation. Cell.

[38] Swartz MA, Iida N, Roberts EW, Sangaletti S, Wong MH, Yull FE (2012). Tumor microenvironment complexity: emerging roles in cancer therapy. Can Res.

[39] Stanta G, Bonin S (2018). Overview on clinical relevance of intra-tumor heterogeneity. Front Med.

[40] Wang M, Zhao J, Zhang L, Wei F, Lian Y, Wu Y (2017). Role of tumor microenvironment in tumorigenesis. J Cancer.

[41] Catalano V, Turdo A, Franco SD, Dieli F, Todaro M, Stassi G (2013). Tumor and its microenvironment: a synergistic interplay. Semin Cancer Biol.

[42] Sormendi S, Wielockx B (2018). Hypoxia pathway proteins as central mediators of metabolism in the tumor cells and their microenvironment. Front Immunol.

[43] Warburg O (1956). On the origin of cancer cells. Science.

[44] Schornack PA, Gillies RJ (2003). Contributions of cell metabolism and H+ diffusion to the acidic pH of tumors. Neoplasia.

[45] Damaghi M, Wojtkowiak JW, Gillies RJ (2013). pH sensing and regulation in cancer. Front Physiol.

[46] Zhang X, Lin Y, Gillies RJ (2010). Tumor pH and its measurement. J Nucl Med.

[47] Chen LQ, Pagel MD (2015). Evaluating pH in the extracellular tumor microenvironment using CEST MRI and other imaging methods. Adv Radiol.

[48] Mahadevan D, Von Hoff DD (2007). Tumor-stroma interactions in pancreatic ductal adenocarcinoma. Mol Cancer Ther.

[49] Vonderheide RH, LoRusso PM, Khalil M, Gartner EM, Khaira D, Soulieres D (2010). Tremelimumab in combination with exemestane in patients with advanced breast cancer and treatment-associated modulation of inducible costimulator expression on patient T cells. Clin Cancer Res.

[50] Vonderheide RH, Domchek SM, Clark AS (2017). Immunotherapy for breast cancer: what are we missing?. Clin Cancer Res.

[51] Sulea T, Rohani N, Baardsnes J, Corbeil CR, Deprez C, Cepero-Donates Y (2020). Structure-based engineering of pH-dependent antibody binding for selective targeting of solid-tumor microenvironment. MAbs.

[52] Mohan N, Jiang J, Dokmanovic M, Wu WJ (2018). Trastuzumab-mediated cardiotoxicity: current understanding, challenges, and frontiers. Antib Ther.

[53] Kaul M, Loos M (1997). Dissection of C1q capability of interacting with IgG: time-dependent formation of a tight and only partly reversible association. J Biol Chem.

[54] López DH, Trevani AS, Salamone G, Andonegui G, Raiden S, Giordano M (1999). Acidic pH increases the avidity of FcγR for immune complexes. Immunology.

[55] Nguyen AW, Liu Y, Maynard J (2018). Enhancing the immunotherapeutic Trastuzumab for selective activity in the low pH tumor microenvironment. J Immunol.

[56] Pilon-Thomas S, Kodumudi KN, El-Kenawi AE, Russell S, Weber AM, Luddy K (2015). Neutralization of tumor acidity improves antitumor responses to immunotherapy. Cancer Res.

[57] Deng J, Li J, Sarde A, Lines JL, Lee Y-C, Qian DC (2019). Hypoxia-induced VISTA promotes the suppressive function of myeloid-derived suppressor cells in the tumor microenvironment. Cancer Immunol Res.

[58] Kuwana Y, Asakura Y, Utsunomiya N, Nakanishi M, Arata Y, Itoh S (1987). Expression of chimeric receptor composed of immunoglobulin-derived V resions and T-cell receptor-derived C regions. Biochem Biophys Res Commun.

[59] Gross G, Waks T, Eshhar Z (1989). Expression of immunoglobulin-T-cell receptor chimeric molecules as functional receptors with antibody-type specificity. PNAS.

[60] Bouchkouj N, Kasamon YL, de Claro RA, George B, Lin X, Lee S (2019). FDA approval summary: axicabtagene ciloleucel for relapsed or refractory large B-cell lymphoma. Clin Cancer Res.

[61] Park JH, Geyer MB, Brentjens RJ (2016). CD19-targeted CAR T-cell therapeutics for hematologic malignancies: interpreting clinical outcomes to date. Blood.

[62] O’Leary MC, Lu X, Huang Y, Lin X, Mahmood I, Przepiorka D (2019). FDA approval summary: tisagenlecleucel for treatment of patients with relapsed or refractory B-cell precursor acute lymphoblastic leukemia. Clin Cancer Res.

[63] Jin Z, Xiang R, Qing K, Li X, Zhang Y, Wang L (2018). The severe cytokine release syndrome in phase I trials of CD19-CAR-T cell therapy: a systematic review. Ann Hematol.

[64] Neelapu SS, Tummala S, Kebriaei P, Wierda W, Gutierrez C, Locke FL (2018). Chimeric antigen receptor T-cell therapy — assessment and management of toxicities. Nat Rev Clin Oncol.

[65] Linette GP, Stadtmauer EA, Maus MV, Rapoport AP, Levine BL, Emery L (2013). Cardiovascular toxicity and titin cross-reactivity of affinity-enhanced T cells in myeloma and melanoma. Blood.

[66] Newick K, Moon E, Albelda SM (2016). Chimeric antigen receptor T-cell therapy for solid tumors. Mol Ther Oncol.

[67] Klebanoff CA, Rosenberg SA, Restifo NP (2016). Prospects for gene-engineered T cell immunotherapy for solid cancers. Nat Med.

[68] Rosenberg SA, Restifo NP (2015). Adoptive cell transfer as personalized immunotherapy for human cancer. Science.

[69] Gerlinger M, Rowan AJ, Horswell S, Larkin J, Endesfelder D, Gronroos E (2012). Intratumor heterogeneity and branched evolution revealed by multiregion sequencing. N Engl J Med.

[70] Sigalotti L, Fratta E, Coral S, Tanzarella S, Danielli R, Colizzi F (2004). Intratumor heterogeneity of cancer/testis antigens expression in human cutaneous melanoma is methylation-regulated and functionally reverted by 5-Aza-2’-deoxycytidine. Can Res.

[71] McGranahan N, Swanton C (2015). Biological and therapeutic impact of intratumor heterogeneity in cancer evolution. Cancer Cell.

[72] Chen YY, Increasing T (2018). Cell versatility with SUPRA CARs. Cell.

[73] Fedorov VD, Themeli M, Sadelain M (2013). PD-1– and CTLA-4–based inhibitory chimeric antigen receptors (iCARs) divert off-target immunotherapy responses. Sci Transl Med.

[74] Sukumaran S, Watanabe N, Bajgain P, Raja K, Mohammed S, Fisher WE (2018). Enhancing the potency and specificity of engineered T cells for cancer treatment. Cancer Discov.

[75] Roybal KT, Rupp LJ, Morsut L, Walker WJ, McNally KA, Park JS (2016). Precision tumor recognition by T cells with combinatorial antigen-sensing circuits. Cell.

[76] Rafiq S, Hackett CS, Brentjens RJ (2020). Engineering strategies to overcome the current roadblocks in CAR T cell therapy. Nat Rev Clin Oncol.

[77] Depil S, Duchateau P, Grupp SA, Mufti G, Poirot L (2020). ‘Off-the-shelf’ allogeneic CAR T cells: development and challenges. Nat Rev Drug Discov.

[78] Majzner RG, Mackall CL (2019). Clinical lessons learned from the first leg of the CAR T cell journey. Nat Med.

[79] Papadopoulou* ANM and LC. CAR T-cell Therapy: A New Era in Cancer Immunotherapy. Current Pharmaceutical Biotechnology 2017;19:5–18. http://www.eurekaselect.com/161365/article accessed March 24, 2020 10.2174/1389201019666180418095526 29667553

[80] Pearce EL, Pearce EJ (2013). Metabolic pathways in immune cell activation and quiescence. Immunity.

[81] Wang R, Green DR (2012). Metabolic checkpoints in activated T cells. Nat Immunol.

[82] Ho P-C, Bihuniak JD, Macintyre AN, Staron M, Liu X, Amezquita R (2015). Phosphoenolpyruvate is a metabolic checkpoint of anti-tumor T cell responses. Cell.

[83] Frost GI, Guibinga GH, Onuffer JJ, Haerizadeh F. Methods and compositions for transducing lymphocytes and regulating the activity thereof. WO2018009923A1, 2018.

[84] Short JM. Conditionally active chimeric antigen receptors for modified T-cells. US20170260261A1, 2017.

[85] Debebe Z, Rathmell WK (2015). Ror2 as a therapeutic target in cancer. Pharmacol Ther.

[86] Zhu C, Wei Y, Wei X (2019). AXL receptor tyrosine kinase as a promising anti-cancer approach: functions, molecular mechanisms and clinical applications. Mol Cancer.

[87] Chaparro-Riggers J, Liang H, DeVay RM, Bai L, Sutton JE, Chen W (2012). Increasing serum half-life and extending cholesterol lowering in vivo by engineering antibody with pH-sensitive binding to PCSK9. J Biol Chem.

[88] Henne KR, Ason B, Howard M, Wang W, Sun J, Higbee J (2015). Anti-PCSK9 antibody pharmacokinetics and low-density lipoprotein-cholesterol pharmacodynamics in nonhuman primates are antigen affinity-dependent and exhibit limited sensitivity to neonatal fc receptor-binding enhancement. J Pharmacol Exp Ther.

[89] Strohl WR (2015). Fusion proteins for half-life extension of biologics as a strategy to make biobetters. BioDrugs.

[90] Qiu Y, Lv W, Xu M, Xu Y (2016). Single chain antibody fragments with pH dependent binding to FcRn enabled prolonged circulation of therapeutic peptide in vivo. J Control Release.

[91] Kang JC, Sun W, Khare P, Karimi M, Wang X, Shen Y (2019). Engineering a HER2-specific antibody–drug conjugate to increase lysosomal delivery and therapeutic efficacy. Nat Biotechnol.

[92] Press Announcements > FDA approves new treatment for a type of late-stage skin cancer n.d. https://web.archive.org/web/20110327063147/https:/www.fda.gov/NewsEvents/Newsroom/PressAnnouncements/ucm1193237.htm accessed March 27, 2020

[93] Zhang Y, Du X, Liu M, Tang F, Zhang P, Ai C (2019). Hijacking antibody-induced CTLA-4 lysosomal degradation for safer and more effective cancer immunotherapy. Cell Res.

[94] Hou TZ, Verma N, Wanders J, Kennedy A, Soskic B, Janman D (2017). Identifying functional defects in patients with immune dysregulation due to LRBA and CTLA-4 mutations. Blood.

[95] Lo B, Zhang K, Lu W, Zheng L, Zhang Q, Kanellopoulou C (2015). Patients with LRBA deficiency show CTLA4 loss and immune dysregulation responsive to abatacept therapy. Science.

[96] Pachter JS, de Vries HE, Fabry Z (2003). The blood–brain barrier and its role in immune privilege in the central nervous system. J Neuropathol Exp Neurol.

[97] Louveau A, Harris TH, Kipnis J (2015). Revisiting the mechanisms of CNS immune privilege. Trends Immunol.

[98] Carson MJ, Doose JM, Melchior B, Schmid CD, Ploix CC (2006). CNS immune privilege: hiding in plain sight. Immunol Rev.

[99] Hawkins BT, Davis TP (2005). The blood–brain barrier/neurovascular unit in health and disease. Pharmacol Rev.

[100] Wong A, Ye M, Levy A, Rothstein J, Bergles D, Searson PC (2013). The blood–brain barrier: an engineering perspective. Front Neuroeng.

[101] Lajoie JM, Shusta EV (2015). Targeting receptor-mediated transport for delivery of biologics across the blood-brain barrier. Annu Rev Pharmacol Toxicol.

[102] Pulgar VM (2019). Transcytosis to cross the blood brain barrier, new advancements and challenges. Front Neurosci.

[103] Cavaco M, Gaspar D, Castanho M, Neves V (2020). Antibodies for the treatment of brain metastases, a dream or a reality?. Pharmaceutics.

[104] Razpotnik R, Novak N, Čurin Šerbec V, Rajcevic U (2017). Targeting malignant brain tumors with antibodies. Front Immunol.

[105] Villaseñor R, Lampe J, Schwaninger M, Collin L (2019). Intracellular transport and regulation of transcytosis across the blood–brain barrier. Cell Mol Life Sci.

[106] Boado RJ, Zhang Y, Wang Y, Pardridge WM (2009). Engineering and expression of a chimeric transferrin receptor monoclonal antibody for blood–brain barrier delivery in the mouse. Biotechnol Bioeng.

[107] Boado RJ, Zhang Y, Zhang Y, Pardridge WM (2007). Genetic engineering, expression, and activity of a fusion protein of a human neurotrophin and a molecular Trojan horse for delivery across the human blood–brain barrier. Biotechnol Bioeng.

[108] Boado RJ, Zhou QH, Lu JZ, Hui EKW (2010). Pharmacokinetics and brain uptake of a genetically engineered bifunctional fusion antibody targeting the mouse transferrin receptor. Mol Pharm.

[109] Pardridge WM, Alzheimer C (2002). Blood–brain barrier drug targeting enables neuroprotection in brain ischemia following delayed intravenous administration of neurotrophins. Molecular and cellular biology of neuroprotection in the CNS.

[110] Zhang Y, Pardridge WM (2006). Blood–brain barrier targeting of BDNF improves motor function in rats with middle cerebral artery occlusion. Brain Res.

[111] Brown VI, Greene MI (1991). Molecular and cellular mechanisms of receptor-mediated endocytosis. DNA Cell Biol.

[112] Paterson J, Webster CI (2016). Exploiting transferrin receptor for delivering drugs across the blood–brain barrier. Drug Discov Today Technol.

[113] Yu YJ, Zhang Y, Kenrick M, Hoyte K, Luk W, Lu Y (2011). Boosting brain uptake of a therapeutic antibody by reducing its affinity for a transcytosis target. Sci Transl Med.

[114] Niewoehner J, Bohrmann B, Collin L, Urich E, Sade H, Maier P (2014). Increased brain penetration and potency of a therapeutic antibody using a monovalent molecular shuttle. Neuron.

[115] Sade H, Baumgartner C, Hugenmatter A, Moessner E, Freskgård P-O, Niewoehner J (2014). A human blood-brain barrier transcytosis assay reveals antibody transcytosis influenced by pH-dependent receptor binding. PLoS ONE.

[116] Shusta EV, Tillotson BJ. pH-dependent antibodies targeting the transferrin receptor and methods of use thereof to deliver a therapeutic agent. US10233252B2, 2019.

[117] Murtaugh ML, Fanning SW, Sharma TM, Terry AM, Horn JR (2011). A combinatorial histidine scanning library approach to engineer highly pH-dependent protein switches. Protein Sci.

[118] Könning D, Hinz S, Grzeschik J, Schröter C, Krah S, Zielonka S, Nevoltris D, Chames P (2018). Construction of histidine-enriched shark IgNAR variable domain antibody libraries for the isolation of pH-sensitive vNAR fragments. Antibody engineering: methods and protocols.

[119] Yang D, Giragossian C, Castellano S, Lasaro M, Xiao H, Saraf H (2017). Maximizing in vivo target clearance by design of pH-dependent target binding antibodies with altered affinity to FcRn. MAbs.

[120] Engler FA, Polli JR, Li T, An B, Otteneder M, Qu J (2018). “Catch-and-Release” anti-carcinoembryonic antigen monoclonal antibody leads to greater plasma and tumor exposure in a mouse model of colorectal cancer. J Pharmacol Exp Ther.

[121] Bonvin P, Venet S, Fontaine G, Ravn U, Gueneau F, Kosco-Vilbois M (2015). De novo isolation of antibodies with pH-dependent binding properties. MAbs.

[122] Irani V, Guy AJ, Andrew D, Beeson JG, Ramsland PA, Richards JS (2015). Molecular properties of human IgG subclasses and their implications for designing therapeutic monoclonal antibodies against infectious diseases. Mol Immunol.

[123] Natsume A, In M, Takamura H, Nakagawa T, Shimizu Y, Kitajima K (2008). Engineered antibodies of IgG1/IgG3 mixed isotype with enhanced cytotoxic activities. Cancer Res.

[124] Kang TH, Jung ST (2019). Boosting therapeutic potency of antibodies by taming Fc domain functions. Exp Mol Med.

[125] Ward ES, Ober RJ (2018). Targeting FcRn to generate antibody-based therapeutics. Trends Pharmacol Sci.

[126] Bruhns P, Jönsson F (2015). Mouse and human FcR effector functions. Immunol Rev.

[127] Huber AH, Kelley RF, Gastinel LN, Bjorkman PJ (1993). Crystallization and stoichiometry of binding of a complex between a rat intestinal Fc receptor and Fc. J Mol Biol.

[128] Martin WL, West AP, Gan L, Bjorkman PJ (2001). Crystal structure at 28 Å of an FcRn/heterodimeric Fc complex: mechanism of pH-dependent binding. Mol Cell.

[129] Vaccaro C, Zhou J, Ober RJ, Ward ES (2005). Engineering the Fc region of immunoglobulin G to modulate in vivo antibody levels. Nat Biotechnol.

[130] Tawfik DS, Chap R, Eshhar Z, Green BS (1994). pH on–off switching of antibody-hapten binding by site-specific chemical modification of tyrosine. Protein Eng Des Sel.

